# Estimated Glomerular Filtration Rate, All-Cause Mortality and Cardiovascular Diseases Incidence in a Low Risk Population: The MATISS Study

**DOI:** 10.1371/journal.pone.0078475

**Published:** 2013-10-16

**Authors:** Chiara Donfrancesco, Simonetta Palleschi, Luigi Palmieri, Barbara Rossi, Cinzia Lo Noce, Fabio Pannozzo, Belinda Spoto, Giovanni Tripepi, Carmine Zoccali, Simona Giampaoli

**Affiliations:** 1 National Centre of Epidemiology, Surveillance and Health Promotion, Istituto Superiore di Sanità, Rome, Italy; 2 Department of Hematology, Oncology and Molecular Medicine, Istituto Superiore di Sanità, Rome, Italy; 3 Azienda Unità Sanitaria Locale (AUSL), Latina, Italy; 4 Epidemiologia Clinica e Patofisiologia delle Malattie Renali e dell’Ipertensione Arteriosa, CNT, IBIM, Reggio Calabria, Italy; Washington Hospital Center, United States of America

## Abstract

**Background:**

Chronic kidney disease (CKD) independently increases the risk of death and cardiovascular disease (CVD) in the general population. However, the relationship between estimated glomerular filtration rate (eGFR) and CVD/death risk in a general population at low risk of CVD has not been explored so far.

**Design:**

Baseline and longitudinal data of 1465 men and 1459 women aged 35-74 years participating to the MATISS study, an Italian general population cohort, were used to evaluate the role of eGFR in the prediction of all-cause mortality and incident CVD.

**Methods:**

Bio-bank stored sera were used to evaluate eGFR at baseline. Serum creatinine was measured on thawed samples by means of an IDMS-calibrated enzymatic method. eGFR was calculated by the CKD-EPI formula.

**Results:**

At baseline, less than 2% of enrolled persons had eGFR < 60 mL/min/1.73m^2^ and more than 70% had a 10-year cardiovascular risk score < 10%. In people 60 or more years old, the first and the last eGFR quintiles (<90 and ≥109 mL/min/1.73m^2^, respectively) were associated to an increased risk for both all-cause mortality (hazard ratio 1.6, 95% confidence interval 1.2-2.1 and 4.3, 1.6-11.7, respectively) and incident CVD (1.6, 1.0-2.4 and 7.0, 2.2-22.9, respectively), even if adjusted for classical risk factors.

**Conclusions:**

These findings strongly suggest that in an elderly, general population at low risk of CVD and low prevalence of reduced renal filtration, even a modest eGFR reduction is related to all-cause mortality and CVD incidence, underlying the potential benefit to this population of considering eGFR for their risk prediction.

## Introduction

Chronic kidney disease (CKD) is recognized as a global public health problem [1,2]. Patients with CKD are far more likely to die, mainly from cardiovascular diseases (CVD), than to develop kidney failure [3]. CKD is also a risk factor for adverse outcomes in other chronic diseases [4]. The incorporation of simple renal biomarkers in the clinical practice for CVD prevention could be accomplished quickly and the question whether screening the general adult population for renal dysfunction would be useful to prevent incident CVD as well as all-causes mortality appears to be a relevant public health issue [5]. 

Several studies investigated whether impaired renal function is associated with increased risk of death or incident cardiovascular events beyond traditional risk factors [6-13]. The majority of these studies found that overt renal dysfunction independently and significantly associates with an increased risk of both mortality and CVD events. However, whether milder forms of renal dysfunction increases CVD and/or death risk remains a much debated issue [14]. In this regard it has to be noted that studies performed so far were conducted in countries at high risk of CVD and/or high prevalence of CKD while, to our knowledge, no studies have been carried out in populations at both low CKD and CVD risk. Countries at low CVD risk could even take greater advantage of investigating the additive value of novel biomarkers to improve the traditional risk models and better stratify persons with low or moderate CVD risk [15]. 

The Italian population has a substantially lower risk for cardiovascular events than Northern European populations and previous observations in the Malattie Cardiovascolari Aterosclerotiche, Istituto Superiore di Sanità (MATISS) cohort fully confirmed the low risk for CVD in a community of central Italy [16]. The present study assesses the relationship between estimated glomerular filtration rate (eGFR) and risk of all-cause mortality and incident CVD of a population at low risk of CVD and low prevalence of reduced renal filtration using longitudinal data and stored biological samples of the MATISS study [17]. 

## Methods

### Study population

Base-line data of the MATISS Study were collected from 1993 to 1996 in 2592 men and 2866 women, aged 20-79 years, randomly selected from the Italian general population stratified by age and sex. The cohort was extracted from the electoral rolls of a geographical area located about 100 km southeast of Rome (central Italy) and comprising four adjacent rural towns (Sezze, Roccagorga, Bassiano, and Priverno). Participation rate was 60%. MATISS study was approved as part of the CUORE Project by Ethical Committee of the Istituto Superiore di Sanità (ISS, the Italian National Institute of Health) on 15 March 2006; the Ethical Committee approved the use of pooled samples for research activity in epidemiological and genetic/genomic studies. Personal data are stored and managed according to the Italian legislative Decree 196/03 and the Code of Conduct to manage personal data for statistic and scientific purposes (2004). Personal data are excluded from the database used for statistical analyses that, instead, includes an unique code for each person. The same codes were used to label the serum samples stored in the biological bank. 

Among MATISS participants, 1465 men and 1459 women with available stored serum samples, free of cardiovascular events and 35-74 years old at baseline were included in the statistical analysis.

### Cardiovascular risk factors and eGFR at baseline

People were invited by letter at the screening centre. Risk factors were collected using standardized procedures published elsewhere [18,19]. 

Biological specimens collected at study baseline were preserved in nitrogen vapours in the biological bank of the National Centre of Epidemiology, Surveillance and Health Promotion of the ISS. For the purpose of this study, stored serum samples were thawed and assayed for creatinine concentration by the Renal Unit of the department of Hematology, Oncology and Molecular Medicine of the ISS. Serum creatinine was measured by means of an enzymatic IDMS-calibrated method (Creatinine Enzymatic Test, Instrumentation Laboratory SpA, Milano, Italy) performed on the automatic analyzer ILAB300plus (Instrumentation Laboratory SpA). The trueness was checked by analyzing the Certified Reference Material from the National Institute of Standards and Technology (Creatinine in Frozen Human Serum, SRM 967a, target value = 0,847 mg/dL). Method imprecision (coefficient of variation, CV), evaluated as pooled standard deviation (SD) of duplicate measurements of real samples, was 2.5% (mean creatinine value = 0.76 mg/dL). The total error of the method, calculated as bias + 1.96* CV, was less than 5%, thus fulfilling the recommendations of the Laboratory Working Group of the National Kidney Disease Education Program [20]. GFR was estimated from serum creatinine concentration according to the CKD-EPI formula [21].

### End-points

All-cause mortality and fatal and non-fatal coronary and cerebrovascular events were considered as end-points and followed-up until December 2004. Major cardiovascular events, in particular myocardial infarction, stroke, revascularization treatment (bypass or angioplasty), sudden death, were identified with record linkage with mortality and hospital discharge registers. Fatal and non-fatal coronary events were validated using MONICA diagnostic criteria [22,23]. Mortality registers and death certificates were used to assess all-cause mortality [24]. Median time of follow-up was 10.3 years both for all-cause mortality and incident major CVD. Persons lost at follow-up were 21 (0.72% of the baseline cohort).

### Statistical analyses

ANOVA models and chi-squared test were used to compare mean values of cardiovascular risk factors and risk conditions prevalence respectively. Pearson correlation analyses were performed to asses correlation among traditional risk factors and eGFR. The 10-years fatal and non-fatal cardiovascular risk score at baseline was based on the CUORE Project risk equation for person aged 35-69 years [25,26]. According to KDOQI guidelines, eGFR values less than 60 mL/min/1.73m^2^ were considered suggestive of CKD [2]. 

Cox proportional hazard models were performed to assess the prediction role of eGFR in relation to risk of all-cause mortality and incident CVD. eGFR was considered as either continuous or categorical variable and models were adjusted for age, gender and traditional risk factors. Systolic and diastolic blood pressure, total and HDL cholesterol, triglycerides, glycemia, body mass index (BMI), hypertension treatment and smoking habit were considered as possible covariates; only significant and non-collinear risk factors were included in models. 

Due to the U shape of the association between eGFR and risk of death or CVD, Cox models including eGFR as continuous variable were performed with the exclusion of persons with eGFR ≥ 109 mL/min/1.73m^2^.

Given the very low number of persons with CKD (less than 2%), subjects were grouped in three classes based on eGFR quintiles (second, third and fourth quintiles were considered the reference group). Due to the interaction between eGFR and age, Cox proportional hazard models were also performed by age class: 35-59 years old and 60-74 years. 

P-value ≤0.05 were considered statistically significant. Statistical analyses were performed using SAS software, release 9.2 (SAS Institute Inc., Cary, North Carolina, USA).

## Results

### Baseline associations

Table 1 shows main baseline characteristics of 1465 men and 1459 women. Mean age was 53 years both in men and women, only 1.0% of men and 1.6% of women had eGFR less than 60 mL/min/1.73m^2^ (range: 37-137 mL/min/1.73m^2^) and only 0.3% had elevated level of serum creatinine (>1.5 mg/dL for men and >1.3 mg/dL for women, range: 0.4-1.8 mg/dL). The CUORE Project 10-year cardiovascular risk score resulted less than 10% for 73% of men and 92% of women. The eGFR was highly correlated with age (r= -0.544 for men and -0.671 for women, p<0.0001). The prevalence of persons with eGFR<60 mL/min/1.73m^2^ was 3% in those 60 years of age or older (eGFR mean±SD: 88.4±11.7 mL/min/1.73m^2^, range: 37-113 mL/min/1.73m^2^) and 0.5% in those with age less than 60 years (eGFR, mean±SD: 102.7±10.9 mL/min/1.73m^2^, range: 39-137 mL/min/1.73m^2^). 

**Table 1:** Baseline descriptive statistics of the MATISS study, men and women, aged 35-74 years, free of previous cardiovascular diseases.

**Variables**		**Men**			**Women**		
		**n**	**mean**	***sd***	**n**	**mean**	***sd***
***Age (years)***		1465	53	11	1459	53	11
***Serum creatinine (mg/dL)***		1465	0.9	0.1	1459	0.7	0.1
***eGFR by CKD-EPI (mL/min/1.73 m^2^)^***		1465	98	12	1459	99	14
***Systolic Blood Pressure (SBP) (mm Hg)***		1464	140.6	21.9	1457	143.9	24.5
***Diastolic Blood Pressure (DBP) (mm Hg)***		1464	86	12	1457	87	13
***Serum Total Cholesterol (mg/dL)***		1463	223.6	43.5	1457	222.4	41.1
***Serum HDL-Cholesterol (mg/dL)***		1463	48	13	1457	53	12
***Serum Triglycerides (mg/dL)***		1463	173.8	130.7	1457	130.2	72.0
***Serum Fasting Glucose (mg/dL)***		1463	93	23	1457	88	25
***Body Mass Index (BMI) (kg/m^²^)***		1460	27.5	3.7	1455	29.1	4.9
***Cigs***°***/Day(CurrentSmokersonly***)		541	17	12	158	10	7
***10-yearcardiovascularrisk(%***)°°		1373	8.2	8.7	1337	3.2	4.4
		**n**	**percent**		**n**	**percent**	
***eGFR by CKD-EPI^ <60 mL/min/1.73 m^2^***		14	1.0		23	1.6	
***Elevated serum creatinine ^§^***		5	0.3		5	0.3	
***CigaretteSmoking***		571	39.0		164	11.3	
***Diabetes***		90	6.2		80	5.5	
***Blood Pressure -- SBP/DBP ^§§^***							
	*Normal*	173	11.8		183	12.6	
	Prehypertension	501	34.2		389	26.7	
	*Hypertension-Stage I*	387	26.4		325	22.3	
	Hypertension-Stage II or ***treated***	403	27.5		560	38.4	
***Hypertension Treatment***		155	10.6		346	23.7	
***Obesity (BMI>=30.0 kg/m^2^)***		308	21.1		567	39.0	
***Primary or secondary school***		1189	81.2		1318	90.4	
***10-yearCVRoftheCUOREProject***							
	*CVR < 10.0%*	996	72.5		1232	92.2	
	CVR 10.0-19.9%	247	18.0		88	6.6	
	*CVR >=20.0%*	130	9.5		17	1.3	

Sd: standard deviation;

^ Estimated glomerular filtration rate (eGFR) by Chronic Kidney Disease Epidemiology Collaboration;

° Cigs is for cigarettes;

°° 10-year cardiovascular risk of the CUORE Project based on age, systolic blood pressure, total and HDL cholesterol, diabetes, smoking habit, treatment for hypertension. It is for men and women 35-69 years old;

§ For men, serum creatinine ≥1.5 mg/dl; for women ≥ 1.3 mg/dl;

§§ *Normal*: Systolic Blood Pressure (SBP) < 120 *AND* Diastolic Blood pressure (DBP) < 80 mmHg, no antihypertensive drug treatment; *Prehypertension*: SBP 121-139 *OR* DBP 81-89 mmHg, no antihypertensive drug treatment; *Hypertension* Stage *I*: SBP 140-159 *OR* DBP 90-99 mmHg, no antihypertensive drug treatment; *Hypertension* Stage *II*: SBP > 160 *OR* DBP > 100 mmHg *OR* antihypertensive drug treatment

The boundaries of eGFR quintiles were 39-89 mL/min/1.73m^2^, 90-96 mL/min/1.73m^2^, 97-101 mL/min/1.73m^2^, 102-108 mL/min/1.73m^2^ and 109-137 mL/min/1.73m^2^. Based on quintiles, eGFR was stratified into three classes: low eGFR (<90 mL/min/1.73m^2^), normal eGFR (90-108 mL/min/1.73m^2^) and high eGFR (≥109 mL/min/1.73m^2^). Individuals in the third group were younger and with a more favourable CVD risk profile at baseline, with the exception of the smoking habit (Table 2). In the high eGFR group, only 5.1% of men and 0.6% of women had a 10-year cardiovascular risk (CVR) ≥ 10% whereas, in the low eGFR group, the percentages of men and women with a CVR ≥ 10% were 43.0% and 19.2%, respectively.

**Table 2:** Baseline descriptive statistics by classes of estimated glomerular filtration rate of the MATISS study, men and women, aged 35-74 years, free of previous cardiovascular diseases.

**Variables**		**Men**										**Women**									
		**eGFR ^ <90** mL/min/1.73 m^2^			90≤eGFR **^ <109** mL/min/1.73 m^2^			**eGFR ^** ≥109 mL/min/1.73 m^2^				**eGFR ^ <90** mL/min/1.73 m^2^			90≤eGFR **^ <109** mL/min/1.73 m^2^			**eGFR ^** ≥109 mL/min/1.73 m^2^			
		**n**	**mean**	***sd***	**n**	**mean**	***sd***	**n**	**mean**	***sd***	**p-value**	**n**	**mean**	***sd***	**n**	**mean**	***sd***	**n**	**mean**	***sd***	**p-value**
***Age (years)***		296	59	11	893	53	9	276	42	6	<0.0001	301	62	9	804	54	9	354	42	5	<0.0001
***Serum creatinine (mg/dL)***		296	1.0	0.1	893	0.8	0.1	276	0.8	0.1	<0.0001	301	0.8	0.1	804	0.7	0.1	354	0.6	0.1	<0.0001
***eGFR by CKD-EPI (mL/min/1.73 m^2^)^***		296	80	9	893	99	5	276	113	5	<0.0001	301	78	11	804	100	5	354	114	4	<0.0001
***Systolic Blood Pressure (SBP) (mm Hg)***		296	145	24	892	141	22	276	133	16	<0.0001	301	154	26	802	145	24	354	132	20	<0.0001
***Diastolic Blood Pressure (DBP) (mm Hg)***		296	86	13	892	86	12	276	86	12	1	301	90	14	802	87	13	354	83	13	<0.0001
***Serum Total Cholesterol (mg/dL)***		296	226	45	891	223	42	276	223	45	0.4552	301	228	41	802	226	41	354	210	39	<0.0001
***Serum HDL-Cholesterol (mg/dL)***		296	48	13	891	48	13	276	45	11	0	301	53	13	802	54	13	354	53	11	0
***Serum Triglycerides (mg/dL)***		296	175	139	891	167	112	276	193	170	0.0164	301	139	77	802	132	70	354	118	71	0.000
***Serum Fasting Glucose (mg/dL)***		296	92	19	891	94	23	276	90	27	0	301	90	25	802	88	25	354	84	24	0
***Body Mass Index (BMI) (kg/m^²^)***		296	28	4	891	28	4	273	27	4	0.0006	300	30	5	801	29	5	354	28	5	<0.0001
***Cigs***°***/Day(CurrentSmokersonly***)		76	14	11	329	17	13	136	20	11	0	19	10	7	74	10	7	65	10	8	1
***10-yearcardiovascularrisk(%***)°°		233	12	11	864	9	9	276	4	4	<0.0001	224	6	6	759	3	4	354	1	1	<0.0001
		**n**	**%**		**n**	**%**		**n**	**%**			**n**	**%**		**n**	**%**		**n**	**%**		
***eGFR by CKD-EPI^ <60 mL/min/1.73 m^2^***		14	5		0	0		0	0		-	23	8		0	0		0	0		-
***Elevated serum creatinine ^§^***		5	1.7		893	100.0		276	100.0		<0.0001	5	1.7		804	100.0		354	100.0		<0.0001
***CigaretteSmoking***		87	29		347	39		137	50		<0.0001	19	6		78	10		67	19		<0.0001
***Diabetes***		19	6.4		59	6.6		12	4.4		0.380	21	7.0		43	5.4		16	4.5		0.377
***Blood Pressure -- SBP/DBP ^§§^***																					
	*Normal*	23	7.8		110	12.3		40	14.5			14	4.7		85	10.6		84	23.7		
	Prehypertension	92	31		294	33		115	42			54	18		199	25		136	38		
	*Hypertension-Stage I*	77	26.0		230	25.8		80	29.0			62	20.6		194	24.2		69	19.5		
	Hypertension-Stage II or ***treated***	104	35		258	29		41	15		<0.0001	171	57		324	40		65	18		<0.0001
***Hypertension Treatment***		61	20.6		84	9.4		10	3.6		<0.0001	119	39.5		195	24.3		32	9.0		<0.0001
***Obesity (BMI>=30.0 kg/m^2^)***		70	24		195	22		43	16		0	137	46		327	41		103	29		<0.0001
***Primary or secondary school***		250	84.5		739	82.9		200	72.5		0.000	282	93.7		744	92.7		292	82.5		<0.0001
***10-yearCVRoftheCUOREProject***																					
	*CVR < 10.0%*	133	57.1		601	69.6		262	94.9			181	80.8		699	92.1		352	99.4		
	CVR 10.0-19.9%	60	26		176	20		11	4			35	16		51	7		2	1		
	*CVR >=20.0%*	40	17.2		87	10.1		3	1.1		<0.0001	8	3.6		9	1.2		0	0.0		<0.0001

Sd: standard deviation;p-values refer to ANOVA for continuous variables and to chi-squared test for categoricalvariables;

^ Estimated glomerular filtration rate (eGFR) by Chronic Kidney Disease Epidemiology Collaboration;

° Cigs is for cigarettes;

°° 10-year cardiovascular risk of the CUORE Project based on age, systolic blood pressure, total and HDL cholesterol, diabetes, smoking habit, treatment for hypertension. It is for men and women 35-69 years old;

§ For men, serum creatinine ≥1.5 mg/dL; for women ≥ 1.3 mg/dL;

§§ *Normal*: Systolic Blood Pressure (SBP) < 120 *AND* Diastolic Blood pressure (DBP) < 80 mmHg, no antihypertensive drug treatment; *Prehypertension*: SBP 121-139 *OR* DBP 81-89 mmHg, no antihypertensive drug treatment; *Hypertension* Stage *I*: SBP 140-159 *OR* DBP 90-99 mmHg, no antihypertensive drug treatment; *Hypertension* Stage *II*: SBP > 160 *OR* DBP > 100 mmHg *OR* antihypertensive drug treatment

### All-cause mortality and incident cardiovascular diseases associations

During the follow-up, 163 and 83 deaths, and 100 and 46 cardiovascular events occurred in men and women, respectively (Table 3). Among the 14 men with CKD only 2 died (one for unspecified chronic heart disease and one for cancer) during follow up and no one experienced major coronary or stroke events; 1 death (unspecified chronic heart disease) and 1 major coronary or stroke event occurred among the 23 women with CKD. Crude mortality rate per 1,000 persons decreased from the first to the third eGFR group: 206, 99 and 51 for men and 116, 51 and 20 for women, as well as crude CVD: 91, 68 and 43 for men and 66 , 30 and 6 for women respectively. 

**Table 3:** Follow-up information by gender of the MATISS study, men and women, aged 35-74 years, free of previous cardiovascular diseases.

		**Men**	**Women**
**All cause mortality**			
	*Number of participants*	1,465	1,459
	*Events*	163	83
	*Persons-years (py)*	14,571	14,338
	*Event/10,000 py*	111.9	57.9
**Incident cardiovascular disease**			
	*Number of participants*	1,465	1,459
	*Events*	100	46
	*Persons-years (py)*	14,309	14,241
	*Event/10,000 py*	69.9	32.3
**Incident coronary disease**			
	*Number of participants*	1,465	1,459
	*Events*	67	18
	*Persons-years (py)*	14,312	14,410
	*Event/10,000 py*	46.8	12.5
**Incident cerebrovascular disease**			
	*Number of participants*	1,465	1,459
	*Events*	37	28
	*Persons-years (py)*	14,467	14,267
	*Event/10,000 py*	25.6	19.6

Since a strong interaction between eGFR and age was found, Cox proportional hazard model analyses were carried out by age classes (34-59 and 60-74 years) (Table 4). In the whole sample, only people with eGFR ≥ 109 mL/min/1.73m^2^ showed a significantly high hazard ratio (HR) in relation to all-cause mortality. Among elderly persons, those with an eGFR value between 90 and 108 mL/min/1.73m^2^ (reference group) resulted having a risk of death 40% lower than those with an eGFR less than 90 mL/min/1.73m^2^ (corresponding to HR 1.6), even when the analysis was adjusted for classical risk factors; in the same age group, an eGFR ≥ 109 mL/min/1.73m^2^ was associated to a risk of death 4 times higher than that of the reference group, and more than 2 times higher than that of the group with eGFR < 90 mL/min/1.73m^2^. No significant association was found for 35-59 years old people.

**Table 4:** Cox model hazard ratios for classes of estimated glomerular filtration rate of the MATISS study, men and women, aged 35-74 years, free of previous cardiovascular diseases.

		**All**						**Age 35-59 years**						**Age 60-74 years**					
		**Age and sex adjusted**			**Adjusted for classical risk factors^**			**Sex adjusted**			**Adjusted for classical risk factors^^**			**Sex adjusted**			**Adjusted for classical risk factors^^**		
		HR	95% confidence interval		HR	95% confidence interval		HR	95% confidence interval		HR	95% confidence interval		HR	95% confidence interval		HR	95% confidence interval	
**All-cause mortality**																			
	eGFR<90 (mL/min/1.73 m^2^)	1.0	0.8	1.4	1.1	0.8	1.5	0.9	0.4	2.0	0.9	0.4	2.1	1.5	1.1	2.0	1.6	1.2	2.1
	90≤eGFR<109(mL/min/1.73 m^2^)	1.0	-	-	1.0	-	-	1.0	-	-	1.0	-	-	1.0	-	-	1.0	-	-
	eGFR≥109 (mL/min/1.73 m^2^)	2.6	1.5	4.4	2.3	1.3	4.0	0.9	0.5	1.6	0.9	0.5	1.6	3.7	1.3	10.0	4.3	1.6	11.7
	Number of persons	2924			2917			2039			2033			885			884		
	Numbers of deaths	246			245			62			62			184			183		
**Incident cardiovascular disease**																			
	eGFR<90 (mL/min/1.73 m^2^)	0.9	0.6	1.3	1.0	0.7	1.5	0.2	0.1	1.0	0.3	0.1	1.1	1.4	1.0	2.2	1.6	1.0	2.4
	90≤eGFR<109(mL/min/1.73 m^2^)	1.0	-	-	1.0	-	-	1.0	-	-	1.0	-	-	1.0	-	-	1.0	-	-
	eGFR≥109 (mL/min/1.73 m^2^)	1.6	0.8	3.1	1.5	0.8	2.9	0.6	0.3	1.2	0.6	0.3	1.3	5.6	1.7	18.1	7.0	2.2	22.9
	Numebr of persons	2924			2917			2039			2033			885			884		
	Numbers of cardiovascular events	146			144			51			50			95			94		

Estimated glomerular filtration rate (eGFR) by Chronic Kidney Disease Epidemiology Collaboration;

HR: hazard ratios; ^All cause mortality models were adjusted for age, gender, current smoking , fasting glycemia, systolic blood pressure.

Incident cardiovascular diseases models were adjusted for age, gender, current smoking , fasting glycemia, systolic blood pressure

^^ All cause mortality models were adjusted for gender, current smoking , fasting glycemia, systolic blood pressure.

Incident cardiovascular diseases models were adjusted for gender, current smoking , fasting glycemia, systolic blood pressure.

Similar results were found for incident of major CVD, although the statistical significance was reached only for the group of elderly people with eGFR ≥ 109 mL/min/1.73m^2^.

By further stratifying the first eGFR class (<90 mL/min/1.73m^2,^) into two groups based on the conventional threshold for CKD (60 mL/min/1.73m^2,^), the individuals with a modestly reduced eGFR ( ≥60 and <90 mL/min/1.73m^2^) still showed a 67% higher risk to die (p= 0.0008) or suffer a CV event (p=0.0165).

These results were confirmed when eGFR was tested as a continuous variable: a significant, independent of classical risk factors, inverse relationship between eGFR and all-cause mortality was found; a similar trend was also found for incident CVD (Table 5). 

**Table 5:** Relation of estimated glomerular filtration rate to prediction of all-cause mortality and cardiovascular events of the men and women, aged 35-74 years, free of previous cardiovascular diseases.

	**All**			**Age 35-59 years**				**Age 60-74 years**				
	**eGFR (mL/min/1.73 m^2^**)			**eGFR (mL/min/1.73 m^2^**)				**eGFR (mL/min/1.73 m^2^**)				
	**Coeff.**	HR at more adverse level*	**p-value**	**Coeff.**	HR at more adverse level*	**p-value**		**Coeff.**	HR at more adverse level*	**p-value**		
***Allcausemortality***												
***Classicalriskfactorsadjusted***	-0.0025	0.97	0.6829	0.0181	1.18	0.3290		-0.0178	0.81	0.0020		
***Incident cardiovascular disease***												
***Classicalriskfactorsadjusted***	-0.0020	0.98	0.8030	0.0262	1.28	0.2063		-0.0162	0.83	0.0451		

Estimated glomerular filtration rate (eGFR) by Chronic Kidney Disease Epidemiology Collaboration;

Due to the U shape of the association between eGFR and risk of death or CVD, Cox models were performed with the exclusion of persons with eGFR ≥ 109 mL/min/1.73m^2^;

* Hazard ratio with level 1 standard deviation (std) higher: all eGFR standard deviation (SD) 11.2, age 35-59 eGFR SD 9.3, age 60-74 eGFR SD 11.6;

All cause mortality models were adjusted for age (except those stratified by age classes), gender, current smoking , fasting glycemia, systolic blood pressure.

Incident cardiovascular diseases models were adjusted for age (except those stratified by age classes), gender, current smoking , fasting glycemia, systolic blood pressure and total cholesterol.

## Discussion

This study shows that, in a general population at low risk to develop CVD and with a low prevalence of reduced renal filtration (only 1.0% of men and 1.6% of women had eGFR less than 60 mL/min/1.73m^2^), elderly individuals with either high or low eGFR show higher mortality rates as compared to those with a normal eGFR. No such difference was found in young people (less than 60 years old). This result is consistent when considering eGFR as a variable either categorical or continuous and, more important, is independent on major traditional risk factors such as smoking habit, glycemia and systolic blood pressure. A similar association emerged also with incident CVD, although the statistical significance was lower.

The association of kidney dysfunction to all cause-mortality in elderly persons is in line with other studies in Scandinavian countries. The second Nord-Trøndelag Health Study (HUNT II), a Norwegian community-based health study, found that reduced kidney function and microalbuminuria are independent risk factors for cardiovascular death particularly in elderly persons among which, by adding renal function indicators, a strong improvement of cardiovascular risk models was found [6]. Data from the Uppsala Longitudinal Study of Adult Men (ULSAM), a Sweden community-based cohort, indicated that in elderly men the simultaneous addition of several biomarkers of cardiovascular and renal abnormalities substantially improves the risk stratification for death from cardiovascular causes [27]. An interpretation of these findings is that, due to the higher prevalence of subclinical cardiovascular damage, the predictive power of traditional risk factors is attenuated in the elderly, thus increasing the relative weight of other risk factors such as eGFR. Our data specifically confirm this hypothesis in a Southern European population at low risk of CVD with a CV mortality about 40% to 80% lower than that in Scandinavian countries.

In our analyses, the association between eGFR and incidence of major CVD, even if present in elderly persons, resulted less significant than for all-cause mortality. Such an evidence could be plausibly explained by the lower statistical power due to the lower number of major cardiovascular events as compared to the number of deaths. 

In line with previous reports [9,10,28-30], we found that the risk of death and incidence of major CVD increased at high eGFR values. This finding may appear at odds with the observation that, as expected, in the baseline analyses people with high levels of eGFR were younger, with lower blood pressure, and with a lower 10-year cardiovascular risk at baseline. Importantly, in our population high eGFR were unrelated to obesity (Table 2). The increased risk associated with high eGFR values is generally ascribed to the fall in serum creatinine due to muscle wasting secondary to ill health. Since a positive association between current smoking and increased eGFR has been also found in the present study as well as in previous reports [31-33], we hypothesize that the smoking habit prime some pathogenic processes which underlie, though on a different time scale, both the increase of eGFR and death/CVD risk. In support of this hypothesis is the evidence that current smoking is truly associated with an increase in GFR when the latter was measured by iohexol clearance in a sample from the general population [34]. 

Some limitations in our study should be mentioned. First, the lack of kidney damage biomarkers: the assessment of micro- and macro-albuminuria together with the calculation of eGFR gives much more information than the assessment of eGFR alone, especially for the interpretation of high eGFR values [35] and a possible underestimation of the prevalence of CKD in the study population. In addition, creatinine clearance measurements using 24-hour urine samples could provide useful information for the assessment of eGFR in persons with exceptional dietary intake (vegetarian diet, creatine supplements) or muscle mass (amputation, malnutrition, muscle wasting), and for checking the need to start dialysis in renal patients [2]. However, the above conditions would concern only a small percentage of persons in a general population study such as the MATISS study. 

Second, we assayed creatinine concentration of serum specimens that had been kept frozen for about 20 years. However, previous literature data show that creatinine levels in serum are stable for at least 25 years at -25°C [36]; moreover, as for our experience, the sample storing time had no apparent effect on the analytical perfomances of the creatinine assay adopted.

Finally, due to the low cardiovascular risk and low prevalence of reduced renal filtration in our cohort, the generalizability of our results to other races and ethnic groups may be limited. However this point represents at the same time one of the strengths of this study since surveys conducted in this kind of populations, while having the unique capability to evidence subtle interactions between early renal dysfunctions and risk to develop CVD or to die, are still lacking in the literature. 

Another strength of the present study is that we used an enzymatic, IDMS-calibrated method to measure serum creatinine, preferred to the widely used Jaffe method because of the fewer assay interferences [37]. 

Finally, a main strength of this study is the soundness of the methods applied to construct and update the MATISS database. Actually, the MATISS cohort is one of the first, longer-studied Italian cohorts used to build the cardiovascular risk chart and scores of the CUORE Project, two tools nowadays used in clinical practice to assess cardiovascular risk in primary prevention of adults in Italy [25,38]. 

## Conclusions

The assessment of CKD is potentially practicable on a population-wide basis as it involves relatively simple blood and urine tests. The present analysis shows for the first time that in an elderly, general population with low cardiovascular risk and low prevalence of reduced renal filtration (<60 mL/min/1.73m^2^), even a modest eGFR reduction is related to all-cause mortality and CVD incidence, underlying the potential benefit to this population of considering eGFR for their risk prediction. Preventive treatment is increasingly offered to elderly persons, but applying current guidelines for primary prevention to this population is problematic because one should treat nearly everyone. Thus, more accurate and complex risk models, including new risk factors such as kidney function indicators, should be developed for this population.

The present data provide a solid basis for future, larger studies aimed to definitely assess whether, in countries with CVD and CKD low risk profiles, screening elderly people for eGFR might significantly improve CVD and mortality risk prediction to justify the screening cost and effort. 
