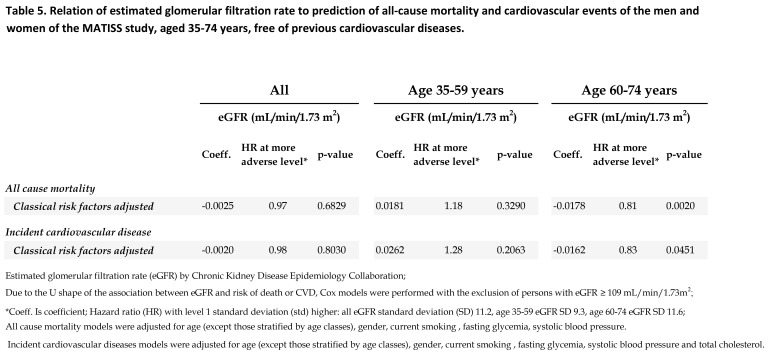# Correction: Estimated Glomerular Filtration Rate, All-Cause Mortality and Cardiovascular Diseases Incidence in a Low Risk Population: The MATISS Study

**DOI:** 10.1371/annotation/1f5e18af-4a68-4419-9f3f-7e8bff410b48

**Published:** 2014-01-15

**Authors:** Chiara Donfrancesco, Simonetta Palleschi, Luigi Palmieri, Barbara Rossi, Cinzia Lo Noce, Fabio Pannozzo, Belinda Spoto, Giovanni Tripepi, Carmine Zoccali, Simona Giampaoli

There are errors in Tables 1-5. 

The correct version of Table 1 can be viewed here: 

**Figure:**
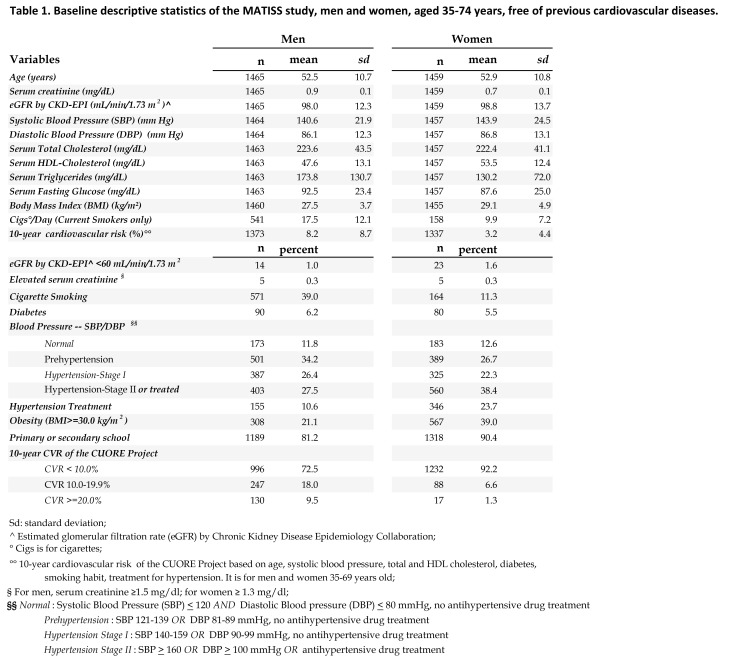


The correct version of Table 2 can be viewed here: 

**Figure:**
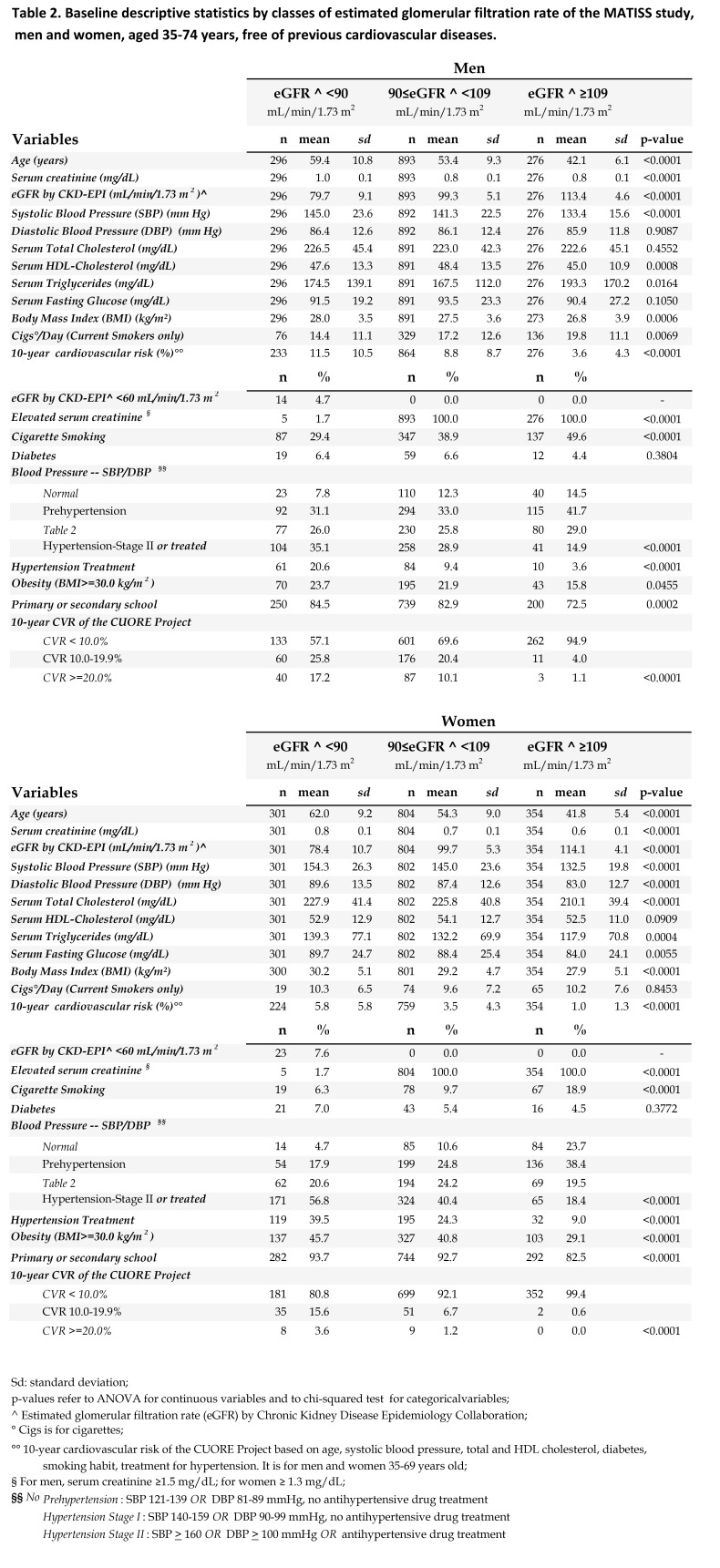


The correct version of Table 3 can be viewed here: 

**Figure:**
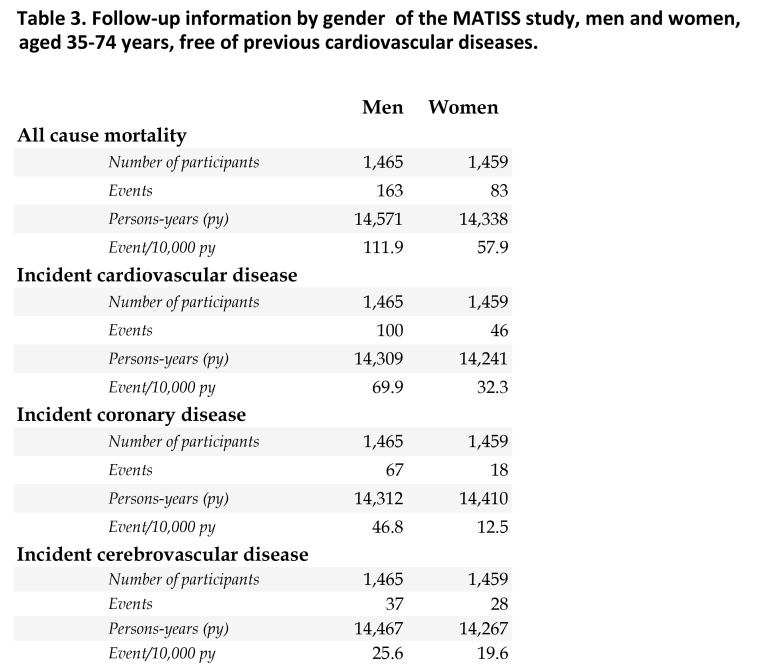


The correct version of Table 4 can be viewed here: 

**Figure:**
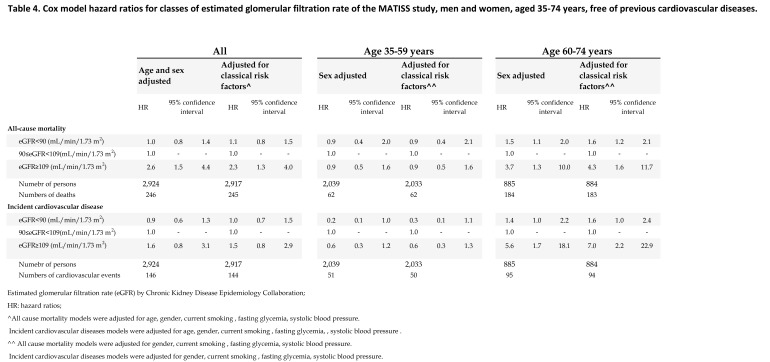


The correct version of Table 5 can be viewed here: 

**Figure:**